# Habitat preference and den characterization of Indian Pangolin (*Manis crassicaudata*) in a tropical lowland forested landscape of southwest Sri Lanka

**DOI:** 10.1371/journal.pone.0206082

**Published:** 2018-11-07

**Authors:** Hasitha Karawita, Priyan Perera, Pabasara Gunawardane, Nihal Dayawansa

**Affiliations:** 1 Department of Forestry and Environmental Science, University of Sri Jayewardenepura, Nugegoda, Sri Lanka; 2 IUCN SSC Pangolin Specialist Group, Zoological Society of London, London, United Kingdom; 3 Department of Zoology and Environment Sciences, University of Colombo, Colombo 07, Sri Lanka; Fred Hutchinson Cancer Research Center, UNITED STATES

## Abstract

The Indian pangolin (*Manis crassicaudata*) is under threat due to hunting for local consumption and illegal trafficking of scales and meat. The dearth of scientific studies on the ecology of the *M*. *crassicaudata* has impaired accurate assessments of its conservation needs. This study investigated the habitat preference and burrow characteristics of *M*. *crassicaudata* in a tropical lowland rainforest in southwest Sri Lanka. A total of 75 burrows (54 feeding burrows and 21 resting burrows) of *M*. *crassicaudata* in four different habitat types i.e. secondary forest, Pine-dominated forest, rubber cultivations and tea-dominated home gardens bordering forest were observed using fixed-width transects in order to characterize resting and feeding burrows of this species. The highest density of resting burrows was recorded from the secondary forest (4ha^-1^), followed by rubber cultivations (2.5ha^-1^) while no resting burrows were recorded in the Pine-dominated forest and the tea-dominated home gardens bordering forest. Feeding burrows were more abundant in the Pine-dominated forest (5.7ha^-1^). The burrow depth, burrow opening height, and width were significantly larger in resting burrows compared to feeding burrows. Resting burrows were located at higher elevations (75-100m) with moderately high slopes (45^0^−60^0^), dense canopy cover (>75%) and away from human habitation. Feeding burrows showed a greater variability in terms of associated environmental features. The study further revealed that Indian pangolins exclusively prefer habitats with rocks and boulders under which they dig resting burrows while the location of feeding burrows largely overlaps with the distribution of prey species. The resting burrow design consisted of a bending tunnel that initially slopes downward and then gradually inclines at an angle between 20 and 30^0^, leading to the resting chamber. Our study highlights the importance of conserving fragmented secondary natural forests in changing landscapes of the southwest lowlands of Sri Lanka as these habitats appear to be critical to sustaining populations of *M*. *crassicaudata*.

## Introduction

The Indian pangolin (*Manis crassicaudata* É. Geoffroy, 1803) is one of the four extant species of pangolin in Asia. Also known as the thick-tailed pangolin, *M*. *crassicaudata* is a medium-sized mammal which is predominantly myrmecophagous and thus, has unique anatomical and behavioral adaptations to prey on ants and termites [1]. Among all Asian pangolin species, the Indian pangolin is arguably the least studied [2] and is the only pangolin species occurring in Sri Lanka.

*M*. *crassicaudata* is known to occur throughout the lowlands of Sri Lanka, from coastal habitats to 1,100m above mean sea level [3]. Its distribution appears to coincide with the range of their main prey species; termites [4]. *M*. *crassicaudata* is of variable abundance in Sri Lanka with only a few known locations in the country where the pangolins are rather frequently encountered or caught by locals [5, 6]. However, no accurate records of their abundance and population size are available, and the species is rarely observed due to its secretive, solitary, and nocturnal habits.

Indian pangolins are fossorial, and they sleep in burrows during the daytime and leave their burrows at night for foraging. The literature suggests that *M*. *crassicaudata* is capable of adapting to a variety of habitats across its geographical range [7]. Habitat features such as tree species composition, vegetation cover and geological features (such as the presence of rock boulders, water sources and soil characteristics) have been identified as important parameters worth considering in the characterization of burrowing habitats of pangolins [2, 5, 8, 9, 10].

Mahmood et al. [11] suggest that the Indian pangolin digs two types of burrows; living or resting burrows and feeding burrows. Resting burrows are used for sleeping/resting during the daytime and breeding while feeding burrows are dug to reach or expose prey species. Parameters such as burrow depth, burrow-opening diameter and the presence of prey remain as well as faecal matter are considered as useful signs to distinguish the two types of burrows [12]. Some reports suggest that a pangolin burrow can have several outlets sealed with loose earth [13], but this may not be the case under all habitat conditions, and remains to be verified by further research. Burrows are usually made under large rocks or boulders and sometimes in the base of trees with the depth of the burrow tending to vary depending on the soil type [14]. Studying the burrow characteristics of *M*. *crassicaudata* in Potohar region of Pakistan, Mahmood et al. [11] concluded that the Indian pangolin usually abandons its resting burrow after a few months of use, and digs a new one within its home range but re-occupy an older resting burrow for up to a year afterward. Feeding burrows, in contrast, are significantly less in depth and have smaller entrances compared to resting burrows [11].

Growing concerns over population declines due to poaching and trafficking have emphasized the need for more concerted conservation efforts for *M*. *crassicaudata* [7, 15]. According to the IUCN Red List of Threatened Species, *M*. *crassicaudata* is listed as ‘Endangered’ due to past and anticipated population declines caused by overexploitation [16]. The species is further included in Appendix I of the Convention on International Trade in Endangered Species of Wild Fauna and Flora (CITES). In Sri Lanka, this species is listed as ‘Near Threatened’ (NT) in the National Red List [17]. *M*. *crassicaudata* is also strictly protected under the Flora and Fauna Protection Ordinance (amendment) Act No. 22 of 2009 of Sri Lanka. However, lack of reliable scientific information on the autecology of *M*. *crassicaudata* remains a major impediment in effective conservation of the species [3].

The Indian pangolin occupies a variety of habitats in its geographical range, and an understanding of its habitat characteristics, habitat preferences and habitat utilization patterns in different environments is vital for better conservation planning of the species. Furthermore, when same habitats are occupied by other burrowing animals such as Greater bandicoot rat (*Bandicota indica*) and Indian crested porcupine (*Hystrix indica*), distinguishing pangolin burrows from those of other animals can be a challenging task for field biologists. Information on habitat preference and den characteristics of *M*. *crassicaudata* is scant in the literature, and the few published studies are highly localized and confined to a single habitat or environment [2, 11, 12]. Habitat utilization and burrow characteristics of Indian pangolins inhabiting tropical rainforest habitats have not been previously studied in detail. Hence, in this study, we examined the key habitat features that influence the habitat preference of *M*. *crassicaudata*, and characterize its resting and feeding burrows in a tropical lowland rainforest associated habitats in the south-west of Sri Lanka.

## Materials and methods

### The study site

**Fig 1:**
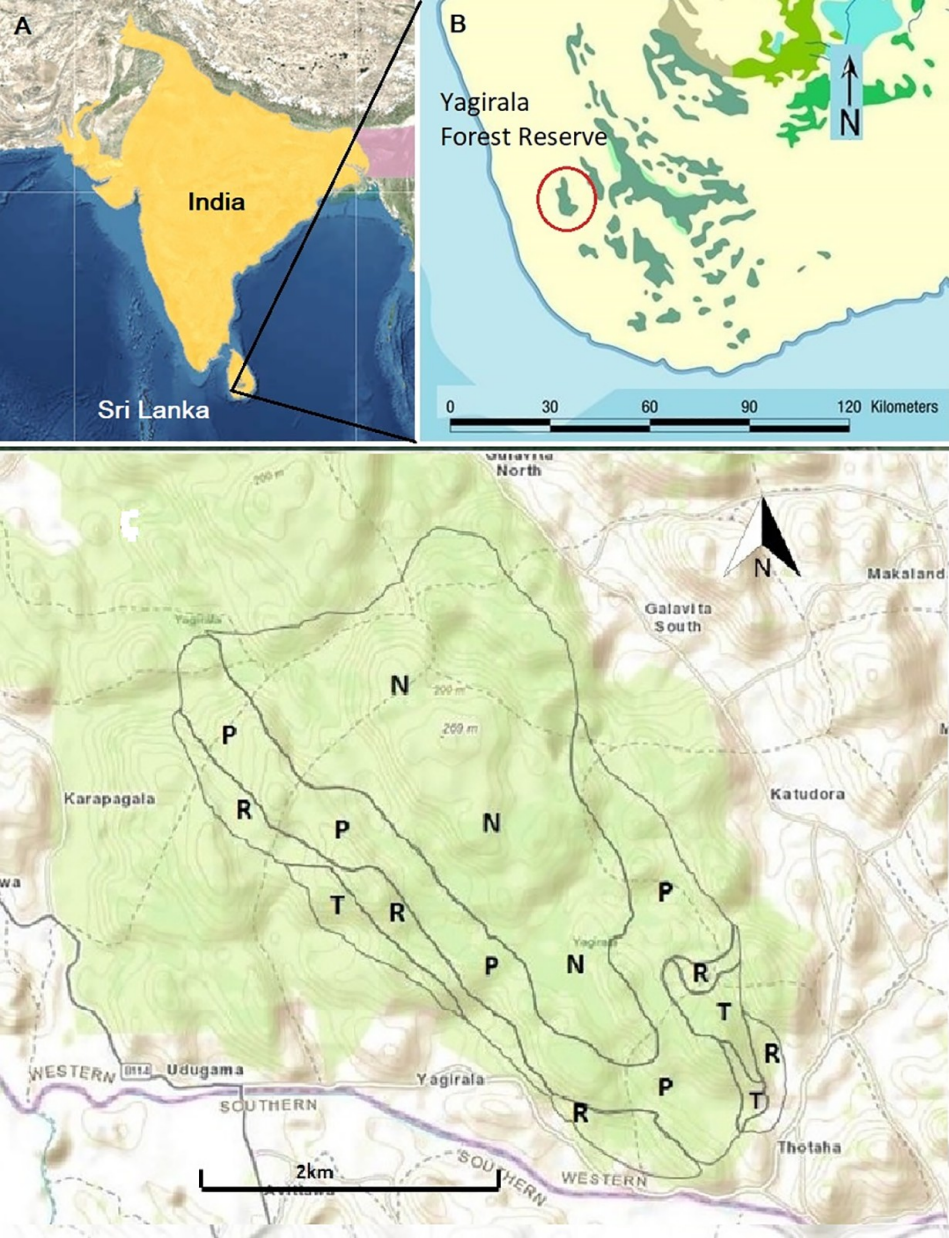
The study site. A: Geographic range of *M*. *crassicaudata* (extant range indicated in yellow) [16] B: The fragmented tropical lowland rainforests (indicated in green) scattered throughout south-west Sri Lanka. C: Distribution of the four habitat types sampled in the Yagirala Forest Reserve (N-Secondary forest; P-Pinus-dominated forest; R-Rubber plantations; T-Tea-dominated home gardens) *Source*: *landsatlook*.*usgs*.*gov*.

This study was conducted in the Yagirala Forest Reserve and its associated habitats, located in the south-west low-country wet zone of Sri Lanka (6°21’ to 6°26’ N and 80°08’ to 80°11’ E). It is a fragmented forest with an area of 2,004.9 ha (Fig 1). The area receives an annual average rainfall of over 3,200 mm during both the north-east monsoons from November to January and the south-west monsoons from May to September. The mean annual temperature ranges from 27.0°C to 30.0°C [18]. The Yagirala forest was selectively logged during the late 1970s and some degraded areas have been replanted with exotic Caribbean pine (*Pinus carribea)*. Agricultural lands such as rubber (*Hevea brasiliensis*), paddy (*Oryza sativa*) and tea (*Camellia sinensis*) dominate homegardens are bordering the Yagirala forest. Hence, field surveys were conducted in four prominent habitat types, namely secondary forest, *Pinus*-dominated forest, rubber plantations, and tea-dominated home-gardens with the total study area extending over 420 ha of accessible area of the forest and adjacent lands (Fig 1C). The main characteristics of the surveyed habitats are summarized in Table 1, adapted and modified from Perera et al. [19]. Rubber plantations and tea-dominated home gardens represent human-modified habitats bordering the forest. Permission was obtained from the Department of Wildlife Conservation, Sri Lanka to carry out the field survey.

**Table 1:** Description of main habitats in the study area.

Habitat type	Description
Secondary forest	Naturally regenerated forest after selective logging operations ceased in 1979. The forest has a canopy cover of 75 to 85% with stratification of vegetation. Canopy height is 25–40 m, dominated by *Dipterocarpus zeylanicus*, *Mesua ferrea*, *Pericopsis mooniana*, *Artocarpus heterophyllus*, and *Artocarpus nobilis*. Sub-canopy (15–25 m), is dominated by *Chaetocarpus castanocarpus*, *Garcinia hermonii*, *Xylopia championi*, *Horsfieldia iriyaghedhi*, and *Myristica dactyloides*. Understory varies from 5–10 m with a sparse shrub layer. Secondary forest accounted for approximately 130 ha of the study area.
*Pinus*-dominated forest	Areas that were heavily logged or cleared for agriculture before the 1970s and restored with exotic *Pinus carribea* during the 1970’s. These restored areas have been left without any management and thus are in the process of being replaced by native tree species (such as *Dillenia retusa*, *Sandoricum koetjape*, *Schumacheria castaneifolia*, *Thottea siliquosa*, and *Coscinium fenestratum*), with a natural death of pines. Canopy cover is 70–75 percent. The undergrowth is dominated by densely grown *Ochlandra stridula* up to 3 m height. *Pinus*-dominated forest covers approximately 200 ha of the study area.
Rubber plantations	Managed *Hevea brasiliensis* (Rubber) monocultivation stands bordering the forest. Trees up to 20m height, and sparse shrub layer composed of weeds and grasses.
Tea-dominated home gardens	Human-modified habitats immediately bordering the forest (forest-home garden interface) with tea (*Camellia sinensis*) as the dominant cultivation. *Gliricidia sepium* trees are maintained at 3–5 m height as shade trees for the tea crop. Multi-purpose and fruit trees in these well-wooded homegardens (*Murraya koenigii*, *Carica papaya*, *Mangifera indica*, *Artocarpus heterophyllus*, *Cocos nucifera*) vary from 2 to 15 m in height.

### Identification and characterization of feeding and resting burrows

Fixed-width line transects were used to survey each habitat for the presence of pangolin burrows. Each transect was 50m in length, and a width of 5m either side to the transect was examined. The placement of transects was dependent on the size of the study habitat, environmental gradient, and accessibility. A minimum distance of 25m was maintained between two adjacent transects to avoid overlapping of sampling areas and to achieve a maximum coverage of each habitat. A total of 300 transects was established in the four selected habitats; 80 transects in the secondary natural forest, 120 transects in the *Pinus-*dominated forest, 40 transects in rubber plantations and 60 transects in tea-dominated home gardens adjoining the forest. The number of transects per habitat type was allocated based on their relative size/area and evidence of the presence of pangolins (eg. burrows, scratch marks on the ground and decaying logs, presence of faecal matter, etc.). Accessibility of the sampling sites and the cost of sampling were further considered in the optimum allocation of samples [20, 21]. As the absence of resting burrows in the *Pinus-*dominated forest was found to be an unusual event, more transacts were placed to make sure that the absence of resting burrows in the *Pinus-*dominated forest is not by chance. Field surveys were conducted between January 2015 and December 2016.

A major issue faced by researchers during the preliminary field observations was the accurate identification of pangolin burrows and distinguishing those from burrows of other animals that occur in the same habitat, particularly the burrows of Indian crested porcupine (*Hystrix indica*). Hence, we relied on a mixed method which utilized both the local knowledge and verifiable evidence to identify pangolin burrows. Before the commencement of transect surveys, we interviewed the local hunters and villagers living adjacent to the forest to identify the burrowing ranges of the Indian pangolins and acquired local knowledge on distinguishing pangolin burrows from those of other animals present in the forest and associated habitats. Accordingly, the presence of footprints, claw marks, faecal samples, scratch marks and shape of the burrow entrance were found to be important clues to identify pangolin burrows accurately. Furthermore, we employed two experienced local villagers/volunteers (as para-biologists) during the initial field surveys in selected areas of the four habitat types to identify the resting and feeding burrows of pangolins. These individuals were employed with their fullest consent after making them aware of the scope of the research. In the context of this study, employing or acquiring information from individuals with local knowledge does not require ethical clearance from an ethical review committee in Sri Lanka. Furthermore, the funding agency for this study (University of Sri Jayewardenepura, Sri Lanka) has a policy of funding only the researches that satisfactorily meet ethical considerations. Based on the nature of the information sought, the funding agency’s Research Review Committee has determined that this research does not require ethical clearance and thus, the funding has been approved.

Those identified as active resting burrows of pangolins (by the visual observations and local knowledge) were further examined with an endoscope (Depstech Digital Endoscope, China) to verify the presence of pangolins inside the burrows. Out of eight active resting burrows inspected, pangolins were present in three burrows, while the other cases could not be verified as it was difficult to reach the resting chamber of the burrow using the endoscope, mainly due to the curvy nature of the burrow entrance.

In each burrow encountered along transects, burrow depth, burrow opening height and width, mid-day temperature and relative humidity inside the burrow were recorded. The burrow features such as burrow depth, burrow opening height and width were measured using a measuring tape while an electronic thermo-humidity meter (OEM-Digital LCD Thermo-Hygrometer, China) was used to measure the temperature and relative humidity inside and outside of the burrow. Humidity and temperature measurements were taken between the time period of 11.00am and 1.00pm.

Opportunistic camera trap surveys were also conducted using eight trail cameras (Browning® Strike Force HD Sub-Micro Series, Missouri, USA). Camera traps were purposefully positioned targeting suspected feeding and resting burrows as well as probable trails leading to the burrows. Such mixed methods have been employed in previous ecological research on pangolins [22, 23, 24].

To examine the design and structure of a resting burrow, four resting burrows appeared to be recently abandoned by the Indian pangolin were excavated carefully using hand tools to expose the cross-section. Based on the measurements of key compartments of the burrow, a generalized cross section was drawn.

### Identification of key habitat features associated with burrows

To assess the habitat features associated with the burrows of Indian pangolins, seven selected environmental features at each burrow site were evaluated. Surveys included measurements of elevation, canopy cover above the burrow, undergrowth associated with the burrow, slope, presence of rock-boulders associated with the burrow, linear distance to the nearest water source and the nearest human settlement. A GPS receiver (Garmin eTrex 30, Hampshire, UK) was used to record the geographic location of the burrow and to measure the elevation of the location of pangolin burrow. Canopy cover was measured using a convex spherical densiometer (Robert E. Lemmon, Bartlesville, Oklahoma, USA). A quadrat of 5x5m was laid to center the burrow opening, and the percentage ground vegetation cover was measured. The linear distance to the closest water source and human settlement from each burrow was measured using Google Earth Pro® software. Preference for a specific habitat feature was determined from the percentage of its occurrence frequencies (P_0_) following Wu et al. [8]. Accordingly, the environmental factors preferred by Indian pangolins were determined and a high value of P_0_ indicated higher preferences for the specific factor.

$$Po=\left(\frac{Frequency\;of\;occurrence\;of\;a\;specific\;habitat\;feature}{Total\;number\;of\;burrows\;observed}\right)\times 100$$

The abundance of pangolin activity in a specific habitat type was determined using a Habitat Preference Index (HPI) computed as HPI = *S*_*n*_ /A where; *S*_*n*_ = total number of pangolin burrows and A = area covered by the fixed-width transect.

The identification of resting and feeding burrows was based predominantly on local knowledge and objective evidence from camera traps and endoscopic observations. Therefore, to statistically distinguish the two types of burrows based on their physical and environmental features, discriminant analysis was used. To perform the discriminant analysis, first, the null hypothesis of “both groups has equal population covariance matrices i.e. variances of both groups are equal” was tested using Box’s test. Based on the canonical discriminant function derived, all cases were classified into feeding and resting burrows. The independent sample t-test was further used to compare the means of selected burrow features/characteristics between resting and feeding burrows. All statistical analysis were performed using the PASW Statistics 18 software package [25].

## Results

**Fig 2:**
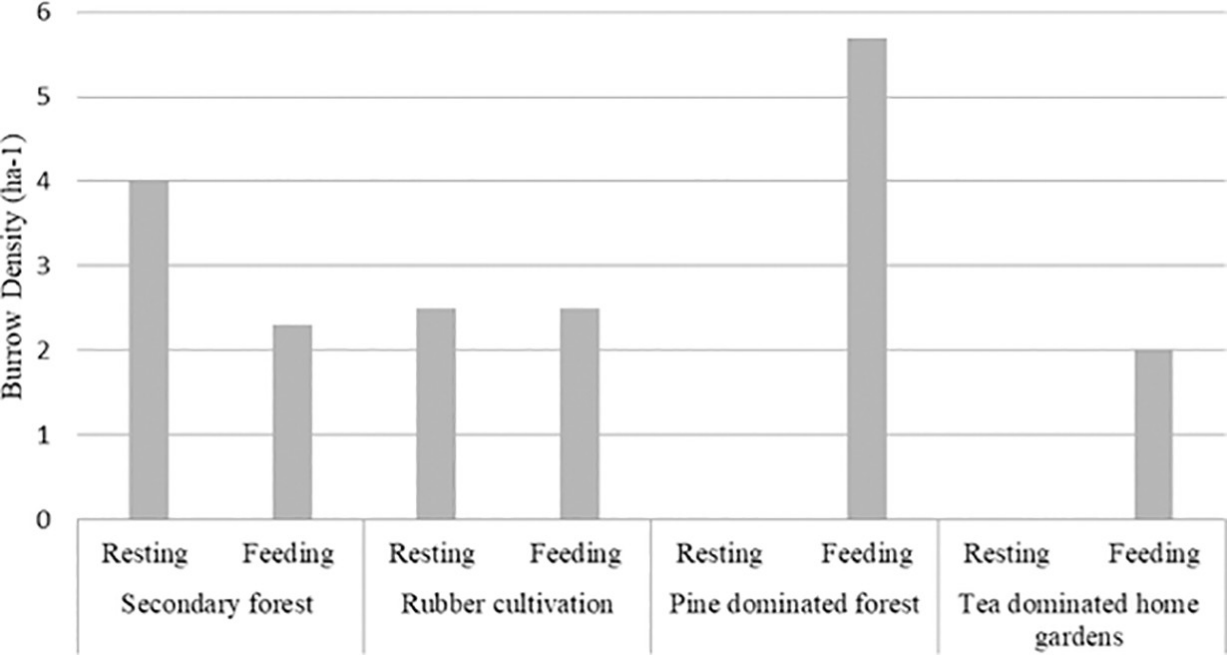
Resting and feeding burrow density in different habitats.

During the study period, a total of 75 burrows was identified. This included 54 feeding burrows and 21 resting burrows. The highest density of resting burrows was recorded from the Secondary forest (4ha^-1^), followed by rubber cultivations (2.5ha^-1^) while no resting burrows were recorded from Pine-dominated forest and tea-dominated forest-home garden interface (Fig 2). In contrast, feeding burrows were more abundant in the Pine-dominated forest (5.7ha^-1^), followed by rubber cultivations (2.5ha^-1^), secondary forest (2.3ha^-1^) and tea-dominated home gardens (2ha^-1^).

Based on the total number of pangolin burrows observed in each habitat type, the HPI values were calculated for the four habitat types. The highest HPI value was recorded for the Pine dominated forest, followed by secondary natural forest, tea dominated forest-home garden interface and rubber plantations (Table 2).

**Table 2:** Number of transects/burrows and the HPI values for the four different habitat types.

Habitat	No. of transects	No: of burrows	Area sampled (ha)	HPI	Rank of preference
Secondary forest	80	25	4	6.3	1
*Pinus*-dominated forest	120	34	6	5.7	2
Rubber plantations	40	10	2	5.0	3
Tea-dominated forest-home garden interface	60	6	3	2.0	4

Selected environment features associated with burrows were identified and measured. The occurrence frequency (P_0_) of each environment feature was also determined (Table 3). The highest number of pangolin burrows were recorded between 56m and 100m elevations under moderate canopy cover. Burrows were more frequent in moderately sloping places with less undergrowth and often associated with rock boulders. Most burrows were observed within 500m from human settlements and 200m from a water source (Table 3).

**Table 3:** The evaluated environment features associated with pangolin burrows.

Environment feature	Description	Subcategory	Number of burrows	P_0_
Elevation		0m~55m	19	25.4%
		56m~75m	24	32.0%
		76m~100m	28	37.3%
		101m~125m	04	5.3%
Canopy closure	Dense	71%~100%	22	29.3%
	Moderate	41%~70%	42	56.0%
	Low	0%~40%	11	14.7%
Undergrowth	Dense	51%~100%	25	33.3%
	Low	0%~50%	50	66.7%
Slope	Low	<30°	38	50.7%
	Moderate	30°~60°	34	45.3%
	Steep	>60°	3	4.0%
Presence of rock boulders	Yes		48	64.0%
	No		27	36.0%
Distance to the nearest water source		0m~100m	27	36.0%
		100m~200m	32	42.7%
		>200m	16	21.3%
Distance to the nearest human settlement		<200m	36	48.0%
		201m~500m	35	46.7%
		>500m	4	5.3%

The discriminant analysis was performed using variables listed in Table 3 (except mid-day temperature and relative humidity inside the burrow). Statistical non-significance for the Box’s test (*p = 0*.*208*) indicated that the assumption of equal population covariance matrices has met to perform the discriminant analysis. The summary of the canonical discriminant function derived is provided in Table 4. A higher eigenvalue suggested that a greater proportion of variance is explained by the discriminant function. The Chi-square statistic to test the significance of Wilk's Lambda (*X*^*2*^ *= 28*.*56*, *p = 0*.*000*) further indicated that the corresponding function explains the group membership satisfactorily.

**Table 4:** Summary of canonical discriminant function.

Function	Eigenvalue	% of Variance	Cumulative %	Canonical correlation
1	0.892^a^	100.0	100.0	0.667

Table 5 shows the results of the classification of resting and feeding burrows using the discriminant model. The derived discriminant function was able to correctly classify 94.6% of original grouped cases.

**Table 5:** Classification of feeding and resting burrows using discriminant analysis.

		Burrow Type	Predicted Group Membership		Total
			Feeding	Resting	
Original	Count	Feeding	51	3	54
		Resting	1	20	21
	%	Feeding	94.4	5.6	100
		Resting	4.8	95.2	100

Subsequent independent-samples t-tests performed to test whether the means of selected burrow features/characteristics would differ between resting and feeding burrows revealed that habitat features such as elevation, slope, canopy cover, and linear distance to the closest human habitation significantly differed between the two burrow types (Table 6). Burrow opening width, height, and depth were significantly higher in resting burrows compared to feeding burrows. However, the environmental parameters of mid-day temperature and relative humidity inside the burrow did not significantly differ between feeding and resting burrows.

**Table 6:** Comparison of burrow characteristics between resting and feeding burrows.

Habitat/burrow characteristic	Resting burrow (n = 21)	Feeding burrow (n = 54)	t	Sig. (p)
Elevation (m)	94.33	64.31	2.77	0.01*
Slope (^o^)	48.38	26.06	8.14	0.00*
Canopy cover (%)	74	59	2.97	0.01*
Undergrowth (%)	55	50	1.83	0.07
Linear distance to the closest water source (m)	119.76	121.37	-0.18	0.86
Linear distance to the closest human habitation (m)	355.62	168.02	2.82	0.01*
Burrow opening width (cm)	46.04	17	3.12	0.01*
Burrow opening height (cm)	59.08	24.12	4.13	0.00*
Burrow depth (cm)	261.12	68.12	10.03	0.00*
Mid-day temperature inside the burrow (^o^C)	28.34	28.66	-1.91	0.07
Mid-day relative humidity inside the burrow (%)	92.71	91.98	0.64	0.53

*Statistical significance at α = 0.05

**Fig 3:**
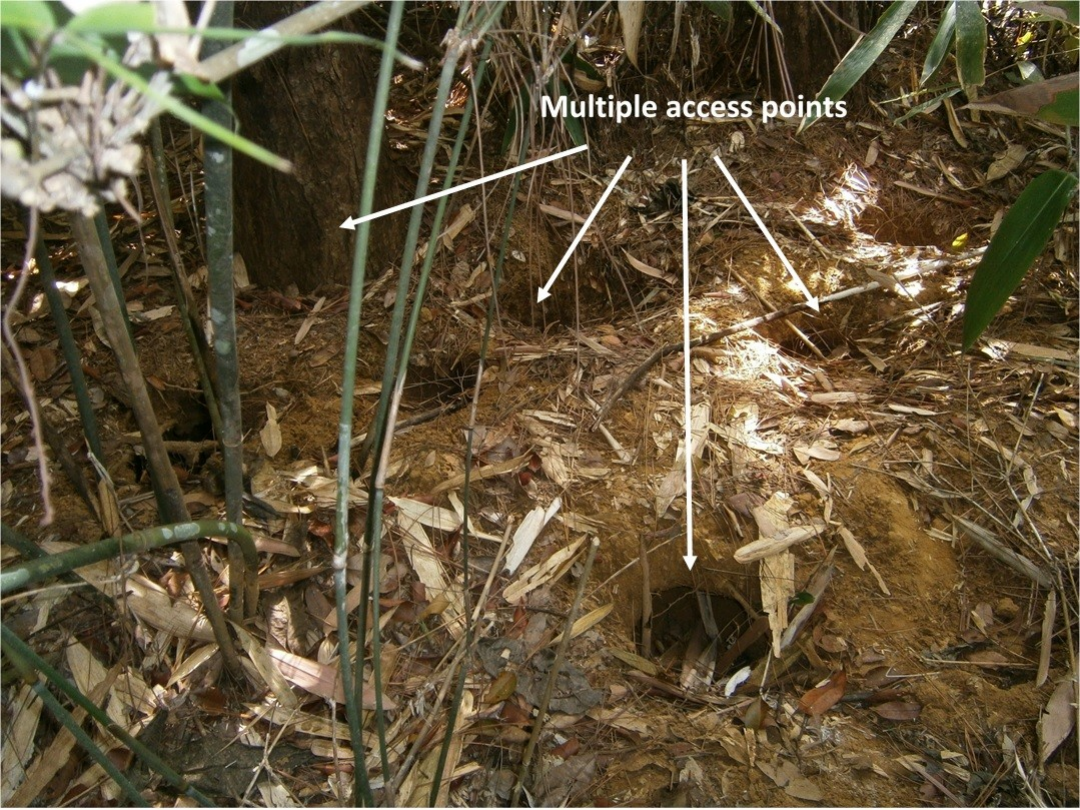
Multiple feeding burrows at the same site leading to a single termite colony.

Most of the feeding burrows were observed as groups, which could be identified as multiple access points leading to a single termite colony (Fig 3). According to the results, approximately 50% of the feeding burrows were recorded in association with rock boulders while the rest was observed on laterite soils with high clay content where termites prefer to build their nests.

**Fig 4:**
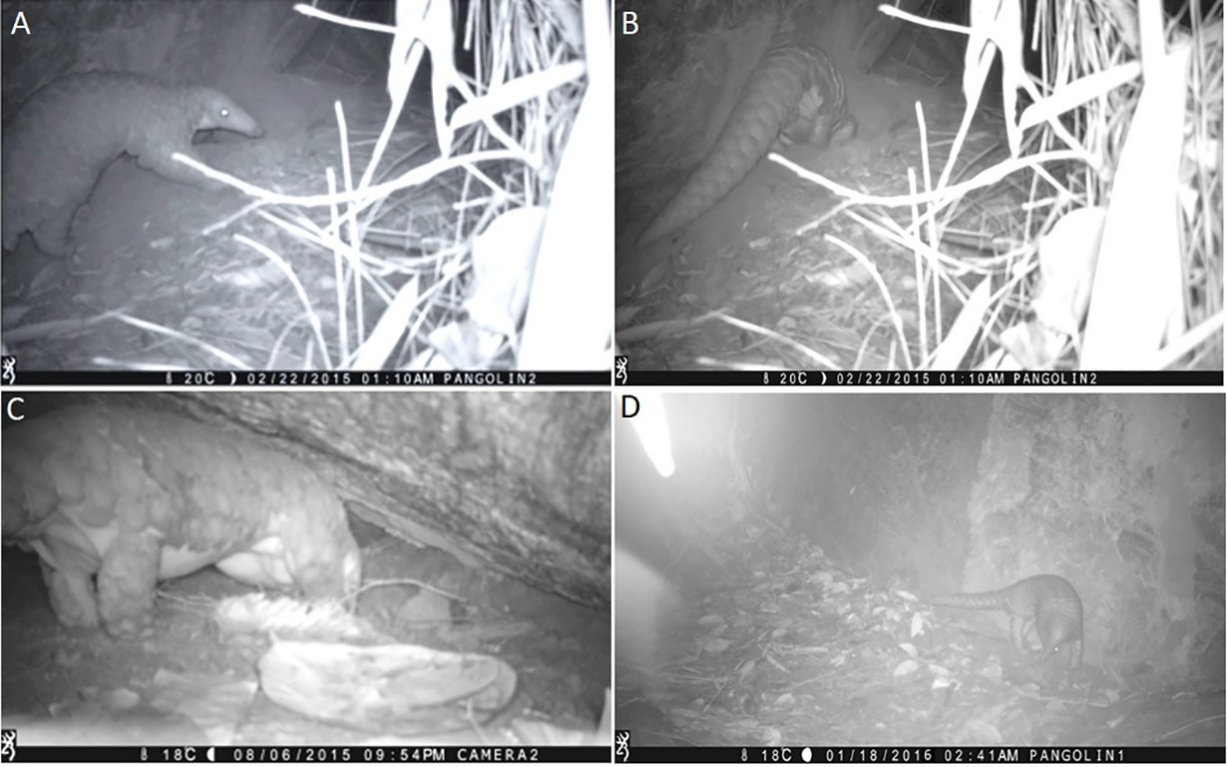
Photographic records of pangolins. Photographs A and B recorded on 22^nd^ February 2015 show an adult pangolin accessing a feeding burrow. Photograph C recorded on 06^th^ August 2015 shows the same feeding burrow being accessed by a pangolin. Photograph D was recorded on 18^th^ January 2016 by a camera trap positioned on a trail leading to a suspected resting burrow.

The photographic evidence from camera trapping at pre-identified feeding burrows confirmed the repetitive use of a feeding burrow after a certain time interval varying between 3 to 5 months (Fig 4). However, it is unknown whether the same individual is repeatedly visiting the feeding burrow where camera traps were positioned.

### The profile of a resting burrow

**Fig 5:**
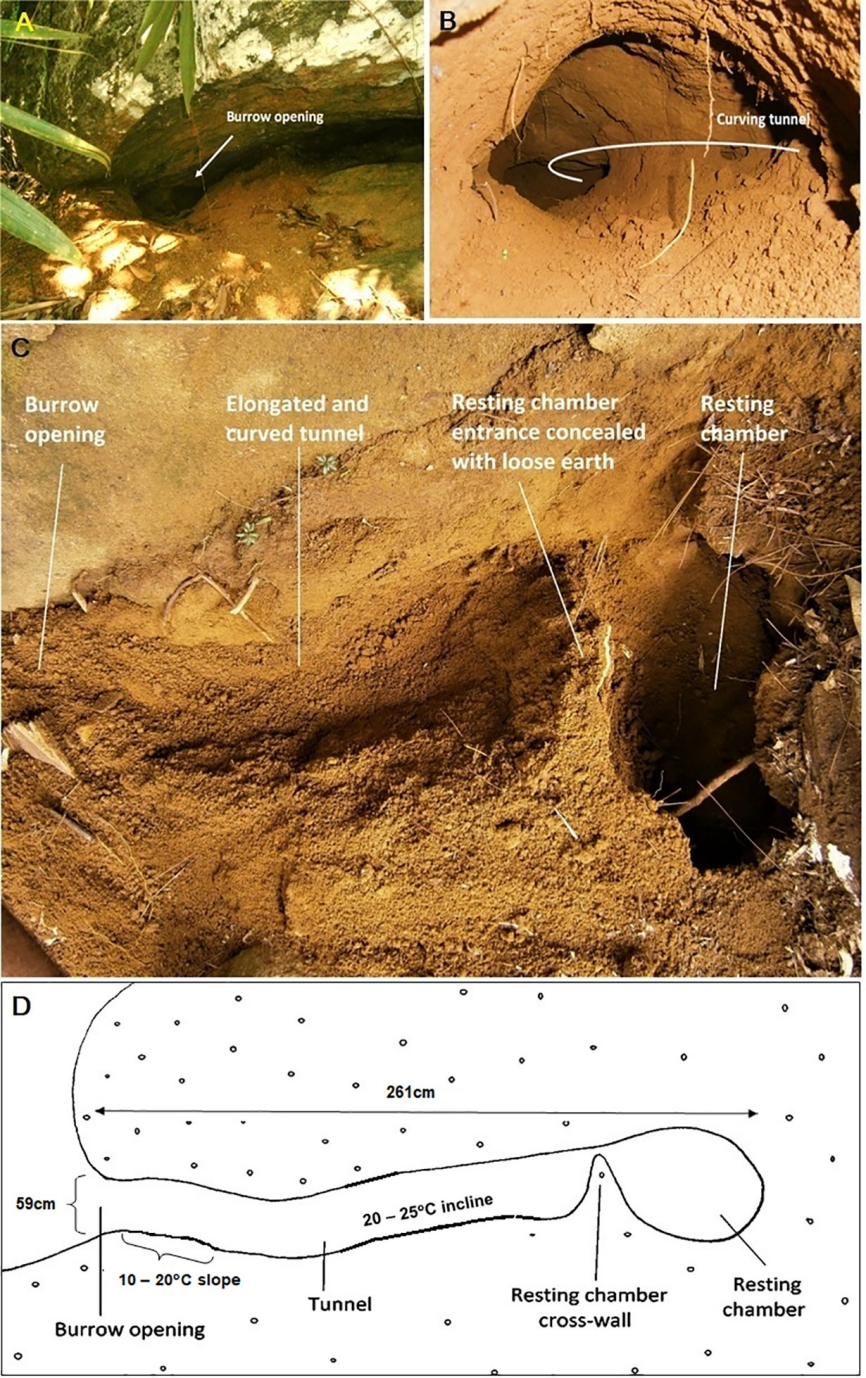
Resting burrow structure. A. Resting burrow located in association with rock boulders; B. The curving entrance tunnel; C. Cross section of a resting burrow; D. Schematic diagram of the cross-section of a resting burrow.

Field observations revealed that all resting burrows were located in association with rock boulders (Fig 5A). The resting burrow design of *M*. *crassicaudata* was found to have a tunnel that first slopes downward approximately 10^0^ to 20^o^ and then inclines about 20^0^ to 25^o^, leading to the resting chamber. The tunnel often bends slightly either to the left or right (Fig 5B). The tunnel leads to the resting chamber and the size of the resting chamber is dependent on the size of the animal. The length of the entrance tunnel is approximately 3 times the length of the resting burrow. The resting chamber entrance is concealed with loose earth (Fig 5C). A schematic diagram of the cross-section of a resting burrow of *M*. *crassicaudata* is illustrated in Fig 5D.

## Discussion

In this study, we investigated the habitat preference and den characteristics of the Indian pangolin inhabiting a fragmented tropical lowland wet evergreen forest in the south-west of Sri Lanka. As pangolins are recorded from agricultural lands and home gardens adjoining the forest, these human-modified habitats were also included in the study. Our results confirm the observations of Mahmood et al. [11] that the Indian pangolin digs out two types of burrows i.e. feeding burrows and resting burrows.

Resting burrows were much deeper and often associated with rock boulders or dug underneath rocks. This strategy may allow extended excavation of the resting burrow without the risk of collapsing due to the lower shear strength of wet soils under frequent rainy conditions in tropical lowland rainforest habitats. Opportunistic observations of 14 resting burrows in three other lowland rainforest patches in the south-west of Sri Lanka by the first author have revealed similar characteristics with respect to the locations of resting burrows. Studying the burrow characteristics of Indian pangolins in the Potohar region of Pakistan, Mahmood et al. [11] reported that resting burrows tend to be associated with buttresses and roots of several tree species. However, these observations were made in areas with less-dense vegetation under arid climatic conditions. Despite the presence of large trees with well-formed buttresses, the Indian pangolins seem to favour rocky substrates to dig their resting burrows in tropical wet forests [5]. As part of an ongoing study, our preliminary observations in dry zone habitats of Sri Lanka revealed that some resting burrows tend to be located at the bases of large trees. This indicates the great adaptability of pangolins to a wide range of environmental conditions.

In this study, we observed that the depth of a resting burrow in the studied habitat can exceed 250cm. This possibly allows greater protection from predators during the daytime while the pangolin is resting. Though there were no natural predators of Indian pangolin recorded in the studied habitats, a major threat was imposed by humans and domestic dogs [6]. In arid habitats of Chakwal District of Potohar Plateau, Pakistan; Mahmood et al. [2] recorded an average resting burrow depth of 143cm, which is significantly shallower compared to our observations. This may suggest that the depth of a resting burrow is dependent on the soil characteristics and geomorphological features [14] where loose wet soils in rainforest may allow the Indian pangolin to dig deeper resting burrows, at the expense of less energy. Mahmood et al. [2] further reported that the average diameter of a resting burrow to be 26.60cm with a characteristic circular shape opening. However, our study recorded an average width and height of 46.04cm and 59.08cm respectively in resting burrows, with more or less elliptic or irregular shaped opening. We observed most resting burrows underneath rock boulders, thus the rock surface often forming one or more sides of the burrow opening. The body shape of the Indian pangolin may further explain the significant difference between burrow opening height and width. Also, the diameter of the burrow opening is dependent on the body size of the pangolin occupying the den. Though some reports suggest that a resting burrow can have several outlets sealed with loose earth [13], we mostly observed resting burrows with a single outlet. However, there was an observation where three resting burrows dug underneath a large rock boulder from different directions, but it could not be verified whether all the three outlets were leading to the same resting chamber.

Feeding burrows, in contrast, were much shallower than resting burrows, with an average depth of 68cm. However, the depth of feeding burrows in our study site was significantly higher than Mahmood et al.’s [2] observations in an arid scrubland habitat where the average depth was 28cm. It is highly likely that feeding burrow depth and other characteristics of a feeding burrow vary on the prey species, geomorphological features of the habitat and terrain. The feeding burrows of the Indian pangolin occurred in groups, often leading to a single termite colony. This strategy possibly increases the feeding efficiency and allows for maximum extraction of prey from the targeted termite colony. Camera trap observations revealed evidence of repeated visits to the same feeding burrow in varying time intervals between 3 to 5 months. The time lag between two consecutive visits probably allows the termite colony to recover, though the frequency of visits to the same feeding sites requires further examination. However, it was not possible to confirm from the photographic records that the repeated visits were made by a single individual occupying the area or different individuals with overlapping home ranges. Such feeding behaviors can be better understood using radiotelemetry techniques [3].

Statistical comparison of feeding and resting burrow dimensions suggests that the time and energy budget for digging resting burrows are comparatively high. The mid-day temperature and relative humidity of the two types of burrows did not differ significantly from each other because both types of burrows were confined more or less to the same soil layer (soil depth); the depth of the resting burrows increased mostly due to burrowing along the horizontal direction rather than vertical direction. The statistical comparison of burrow-associated features further revealed that habitat parameters such as elevation, slope, canopy cover, and linear distance to the closest human habitation from the burrow are significantly higher with resting burrows compared to feeding burrows. Resting burrows were located in higher elevations (75-100m) with moderate slopes (45^0^−60^0^) under dense canopy cover (>75%), and away from human habitations (200-400m). However, the associated environmental features such as canopy closure, undergrowth cover and elevation are likely to be site specific or habitat specific for pangolins. These burrow-associated environmental features show a substantial variability across the range of *M*. *crassicaudata* [2, 3, 5, 11]. Studies on burrow characteristics and habitat preferences of the Chinese pangolin (*M*. *pentadactyla*) across its range countries have also reported variable findings [8, 24, 26]. Feeding burrows, in contrast, showed a greater variability in terms of associated environmental features, even being recorded from home gardens.

Wu et al. [8] observed that *M*. *pentadactyla* digs resting burrows away from sources of interference (human habitation), suggesting an inclination to avoid disturbances. Our study unveiled similar observations for *M*. *crassicaudata* where the linear distance to the closest human habitation was found to be significantly greater for resting burrows while feeding burrows were observed even near households in the forest home garden interface. No significant association with the linear distance to the closest water source and the location of resting and feeding burrows were observed, possibly due to the abundant water sources in tropical lowland rainforest habitat. The highest density of resting burrows was recorded from the secondary forest followed by rubber cultivations, owing to the presence of preferred habitat features to construct resting burrows. The rubber cultivations surveyed in this study included sloping lands with rock boulders, and typically having a dense canopy cover. It was further observed that the resting burrows recorded from rubber cultivations tend to be located away from households adjoining rubber cultivations. No resting burrows were recorded from the *Pinus* dominated forest and the tea-dominated forest-home garden interface. Though there were no remarkable differences in terrain conditions between the secondary forest and *Pinus* dominated forest, the relatively open canopy conditions and the dense *Ochlandra stridula* (Forest bamboo) undergrowth may have discouraged pangolins from constructing resting burrows in *Pinus* dominated forest. No records of resting burrows from the tea-dominated forest-home garden interface suggest that pangolins avoid such habitats due to the lack of preferred habitat features, and also the perceived risk of predation and disturbances by humans and domestic animals such as dogs [6].

The *Pinus* dominated forest and home gardens in the interface were predominantly used as foraging sites. Abundant availability of decaying pine logs in the Pine-dominated forest provide excellent habitats for termite activity and this may explain the use of Pine-dominated forest habitat by pangolins, predominantly as foraging sites. Home gardens in the interface were also used by pangolins for foraging, probably due to the availability of a variety of alternative food types/items. It is reported by local people that Indian pangolins sometimes consume items other than ants and termites (e.g. dried anchovies, cooked rice particles and maggots in waste bins) available in home garden environments, thus showing an omnivorous diet [6].

Trageser et al. [24] studied the burrow design of *M*. *pentadactyla* and reported that resident burrow design consists of a 30 degree upward sloping tunnel, leading to a resting burrow chamber, which is concealed by a false earth wall constructed by the pangolin to protect it from predators. According to our findings, the resting burrow design of *M*. *crassicaudata* differs substantially from *M*. *pentadactyla* because it initially slopes downward (10-20^o^) and then inclines at 20-30^o^ along the tunnel to the resting chamber. Digging a slightly inclining tunnel may be a strategy to prevent water entering the resting chamber during heavy rains as well as to evade predators. Further, the resting chamber entrance was found to be concealed with loose earth rather than the entrance of the burrow as reported in the literature [27].

In this study, the data were collected from a 420ha area of the Yagirala forest. This is a significant proportion of the entire forest and represents the major vegetation/habitat types found in Yagirala forest. The study was focused on four major habitat types within and around the Yagirala forest which were unequal in their extents. Therefore, sampling effort exerted on each habitat was different and the allocation of transects for each habitat was dependent on the extent/area of habitat, accessibility, and sampling cost [20, 21]. Sampling designs partially based on convenience, such as locating sampling sites following access roads, etc. [19, 28], may suffer from unknown biases [29]. However, sampling issues are often difficult to overcome in ecological studies, and ecologists frequently employ such sampling strategies when the primary emphasis is on generalizability (i.e., ensuring that the knowledge gained is representative of the population from which the sample was drawn), despite limited resources, time and workforce [30]. Nonetheless, replicating the study in other tropical lowland forest patches in the south-west and associated habitats would yield more generalizable findings.

## Conclusion

Our study findings suggest that although *M*. *crassicaudata* is capable of occupying natural and man-made habitats, less-disturbed secondary forest is the most important habitat for the species in tropical lowland wet forests of southwestern Sri Lanka. The secondary forest is highly favored by *M*. *crassicaudata* in which to construct resting burrows. Pine-dominated forest and human-modified habitats provide important foraging sites for *M*. *crassicaudata*. Burrow opening width, burrow opening height and burrow depth are useful physical measures for distinguishing resting burrows from feeding burrows. Resting burrows were frequently associated with rock boulders, elevations of 75-100m with moderate slopes (45^0^−60^0^), dense canopy cover (>75%), and distance from human habitations (200-400m). Feeding burrows often occur in groups providing multiple access points to a single termite colony and their distribution was predominantly determined by the occurrence of prey species of *M*. *crassicaudata*. The resting burrow design was found to have a tunnel that first slopes downward approximately 10-20^o^ and then gradually inclines at an angle between 20 and 30^0^, leading to the resting chamber. The entrance tunnel often bends slightly either to the left or right and the resting chamber entrance is concealed with loose earth.

Findings of this study stress the importance of conserving the fragmented secondary natural forest patches which appear to be core habitat that sustains populations of *M*. *crassicaudata* in changing landscapes in the southwest lowlands of Sri Lanka. Human modified habitats in the forest-village interface provide valuable foraging sites for *M*. *crassicaudata*. However, the tendency of *M*. *crassicaudata* to use such habitats can increase its vulnerability to poaching and other risks such as roadkill and attacks from domestic dogs. Hence, there is a need for an integrated conservation strategy for *M*. *crassicaudata*, including a strong community awareness and collaboration component.

## Supporting information

S1 Table Burrow characteristics of resting and feeding burrows. (XLSX)

## References

[1] Yang CW, Chen S, Chang CY, Lin MF, Block E, Lorentsen R, et al History and dietary husbandry of pangolins in captivity. Zoo Biology. 2007 5 1;26 (3):223–30. 10.1002/zoo.20134 19360575

[2] Mahmood T, Irshad N, Hussain R. Habitat preference and population estimates of Indian pangolin (Manis crassicaudata) in District Chakwal of Potohar Plateau, Pakistan. Russian journal of ecology. 2014 1 1; 45(1):70–5. 10.1134/S1067413614010081

[3] Perera PKP, Karawita KV, Pabasara MG. Pangolins (Manis crassicaudata) in Sri Lanka: A Review of Current Knowledge, Threats and Research Priorities. Journal of Tropical Forestry and Environment. 2017 7 30; 7(1):1–14.

[4] Phillips WW. Manual of the mammals of Sri Lanka. Wildlife and Nature Protection Society of Sri Lanka; 1981.

[5] Pabasara MG, Perera PK, Dayawansa NP. Preliminary Investigation of the Habitat Selection of Indian Pangolin (Manis crassicaudata) in a Tropical Lowland Forest in South-West Sri Lanka. Proceedings of International Forestry and Environment Symposium, 2015; 20(4).

[6] Karawita KV, Perera PK, Pabasara MG. Indian Pangolin (Manis crassicaudata) in Yagirala Forest Reserve Ethnozoology and Implications for Conservation. In Proceedings of International Forestry and Environment Symposium, 2016; 21(34).

[7] Chakkaravarthy QA. Research and conservation needs of the Indian pangolin (Manis crassicaudata). InProceedings of Third Seminar on Small Mammals Issues 2012 5 18; 50–55.

[8] Wu SB, Liu NF, Ma GZ, Xu ZR, Chen H. Habitat selection by Chinese pangolin (Manis pentadactyla) in winter in Dawuling Natural Reserve. Mammalia. 2003 1 1;67(4):493–502.

[9] Bhandari N, Chalise MK. Habitat and distribution of Chinese pangolin (Manis pentadactyla Linnaeus, 1758) in Nagarjun Forest of Shivapuri Nagarjun National Park, Nepal. Nepalese Journal of Zoology. 2014;2:18–25.

[10] Mahmood T, Andleeb S, Anwar M, Rais M, Nadeem MS, Akrim F, et al Distribution, Abundance and vegetation analysis of the Scaly Anteater (Manis crassicaudata) in Margalla Hills National Park Islamabad, Pakistan. The Journal of Animal & Plant Sciences. 2015 10 1; 25(5):1311–21.

[11] Mahmood T, Jabeen K, Hussain I, Kayani AR. Plant species association, burrow characteristics and the diet of the Indian pangolin, Manis crassicaudata, in the Potohar plateau, Pakistan. Pakistan Journal of Zoology. 2013 12 1;45(6):1533–9.

[12] Irshad N, Mahmood T, Hussain R, Nadeem MS. Distribution, abundance and diet of the Indian Pangolin (Manis crassicaudata). Animal Biology. 2015 3 3; 65(1):57–71. 10.1163/15707563-00002462

[13] Prater SH. The book of indian animals bombay natural history society and oxford university press. 1980

[14] Prater SH. The book of Indian animals. Bombay natural history society; 1965 (Vol. 2).

[15] Challender DW. Asian pangolins: increasing affluence driving hunting pressure. Traffic Bulletin. 2011;23(3):92–3.

[16] Baillie J, Challender D, Kaspal P, Khatiwada A, Mohapatra R, Nash H. Manis crassicaudata. The IUCN Red List of Threatened Species. 2014 10.2305/IUCN.UK.2014-2.RLTS.T12761A45221874.en

[17] Weerakoon DK. The taxonomy and conservation status of mammals in Sri Lanka. The National Red List 2012 of Sri Lanka. 2012:134.

[18] Punyawardena BVR, Bandara TMJ, Munasinghe M AK, Banda NJ, & Pushpakumara SMV. Agro-ecological regions of Sri Lanka. Natural Resource Management Centre, Department of Agriculture, Peradeniya, Sri Lanka. 2003.

[19] Perera P, Wijesinghe S, Dayawansa N, Marasinghe S, Wickramarachchi C. Response of tropical birds to habitat modifications in fragmented forest patches: A case from a tropical lowland rainforest in south-west Sri Lanka. Community Ecology. 2017 8;18(2):175–83. 10.1556/168.2017.18.2.7

[20] Cochran WG. Sampling Techniques: 3d Ed. New York: Wiley; 1977.

[21] Sutherland WJ, editor. Ecological census techniques: a handbook Cambridge University Press; 2006 8 3.

[22] Newton P, Van Thai N, Roberton S, Bell D. Pangolins in peril: using local hunters’ knowledge to conserve elusive species in Vietnam. Endangered Species Research. 2008 9 23;6(1):41–53. 10.3354/esr00127

[23] Nash HC, Wong MH, Turvey ST. Using local ecological knowledge to determine status and threats of the Critically Endangered Chinese pangolin (Manis pentadactyla) in Hainan, China. Biological Conservation. 2016 4 1; 196:189–95. 10.1016/j.biocon.2016.02.025

[24] Trageser SJ, Ghose A, Faisal M, Mro P, Rahman SC. Pangolin distribution and conservation status in Bangladesh. PloS one. 2017 4 7;12(4):e0175450 10.1371/journal.pone.0175450 28388644 PMC5384767

[25] SPSS I. PASW statistics 18 Chicago: SPSS Inc. 2009.

[26] Thapa P, Khatiwada AP, Nepali SC, Paudel S. Distribution and conservation status of Chinese pangolin (Manis pentadactyla) in Nangkholyang VDC, Taplejung, eastern Nepal. American Journal of Zoological Research. 2014;2(1):16–21. doi: 10.12691/ajzr-2-1-3

[27] Nowak RM. Walker's Mammals of the World JHU Press; 1999 4 7.

[28] Bart J, Hofschen M, Peterjohn BG. Reliability of the Breeding Bird Survey: effects of restricting surveys to roads. The Auk. 1995 7 1:758–61.

[29] Albert CH, Yoccoz NG, Edwards TC Jr, Graham CH, Zimmermann NE, Thuiller W. Sampling in ecology and evolution–bridging the gap between theory and practice. Ecography. 2010 12; 33(6):1028–37.

[30] Etikan I, Musa SA, Alkassim RS. Comparison of convenience sampling and purposive sampling. American Journal of Theoretical and Applied Statistics. 2016 1; 5(1):1–4.

